# Closure simulation for reduction of emergency patient diversion: a discrete agent-based simulation approach to minimizing ambulance diversion

**DOI:** 10.1186/s40001-018-0330-0

**Published:** 2018-06-08

**Authors:** D. Pförringer, M. Breu, M. Crönlein, R. Kolisch, K.-G. Kanz

**Affiliations:** 1 Klinik und Poliklinik für Unfallchirurgie, Klinikum rechts der Isar, Technische Universität München, Ismaninger Str. 22, 81675 Munich, Germany; 2 TUM School of Management, Technische Universität München, Arcisstr. 21, 80333 Munich, Germany

**Keywords:** Emergency medical services, Ambulance, Diversion, Dispatch, Crowding, Closure policy, Simulation

## Abstract

**Background:**

The city of Munich uses web-based information system IVENA to promote exchange of information regarding hospital offerings and closures between the integrated dispatch center and hospitals to support coordination of the emergency medical services. Hospital crowding resulting in closures and thus prolonged transportation time poses a major problem. An innovative discrete agent model simulates the effects of novel policies to reduce closure times and avoid crowding.

**Methods:**

For this analysis, between 2013 and 2017, IVENA data consisting of injury/disease, condition, age, estimated arrival time and assigned hospital or hospital-closure statistics as well as underlying reasons were examined. Two simulation experiments with three policy variations are performed to gain insights on the influence of diversion policies onto the outcome variables.

**Results:**

A total of 530,000+ patients were assigned via the IVENA system and 200,000+ closures were requested during this time period. Some hospital units request a closure on more than 50% of days. The majority of hospital closures are not triggered by the absolute number of patient arrivals, but by a sudden increase within a short time period. Four of the simulations yielded a specific potential for shortening of overall closure time in comparison to the current status quo.

**Conclusion:**

Effective solutions against crowding require common policies to limit closure status periods based on quantitative thresholds. A new policy in combination with a quantitative arrival sensor system may reduce closing hours and optimize patient flow.

## Background

The concept of *ambulance diversion* as a method to relieve emergency department (ED) crowding was first described by Lagoe et al. in 1990 [1]. Patients with minor injuries were diverted to other hospitals with less-crowded EDs. Many countries nowadays have distinctive rules and guidelines defining when and for how long an ED is allowed to go on diversion status [2]. Systems facilitating information exchange regarding treatment possibilities and available capacities between and within hospitals and emergency medical services (EMS) are emerging such as, e.g., capacity command centers. An Australian case study implementing an “*Emergency Department System Viewer*”, a near-real-time display of crowding conditions and incoming emergency patients displayed in the nurse’s station, resulted in a decrease in diversion of 36% [3].

In the state of Bavaria, the Bavarian law on emergency service (“*Bayerisches Rettungsdienstgesetz*”, short BayRDG [4]) regulates emergency transport. The destination decision of the ambulance dispatch center for the next appropriate and available facility has to happen in the best interest of the patient in respect to his condition, potentially resulting in not heading to the closest hospital but to the hospital with suitable facilities instead (e.g., trauma room, stroke unit).

Ambulance diversion has long been discussed and researched within the emergency medicine community. During a survey of directors of emergency departments in California, 96% of the interviewees reported overcrowding as a problem; 28% reported daily overcrowding [5]. A study by Burt et al. showed around 45% of all EDs in the US being on diversion status at least once a year and one ambulance being diverted every minute across the US [6]. Fatovich et al. found diversion hours in Australia to have risen by 75% between 2001 and 2002 [7]. Pham et al. reviewed several studies on diversion times and identified peak times for ambulance diversion to occur on Mondays, afternoons to evenings, winter time and during influenza season [8–10]. Extensive staffing and further economic efforts are invested into limiting or even ending ambulance diversion [11]. As described in earlier research, technological progresses such as telecommunications and informatics bring numerous chances, challenges and problems for medicine [12].

The underlying study for the first time in this format describes the current status in a 1.4 million city in Germany by analyzing more than half a million IVENA recordings. This analysis is followed by an innovative discrete agent-based simulation of the effects of closure policies on the diversion equilibrium. Within the simulation model, a specific set of closure policies and their effects on overall closure times as well as diversion numbers are being analyzed and the subsequent results described. The key hypothesis is to develop a set of policies to reduce closure times and numbers of diverted patients.

## Methods

The city of Munich uses web-based information system IVENA to support coordination of the EMS. IVENA informs about all currently available treatment possibilities. Its use was initiated in February 2013 and since then it continuously captures and dispenses information on 10,000 hospital beds, intensive care and intervention facilities in 40 hospitals in Munich, Germany’s third largest city with 1.5 million inhabitants [13]. IVENAD offers a web-based continuously refreshing, updating and loading surface allowing medical professionals an overview of current transports as well as basic anonymous patient information. Dependent on users’ rights and access authorization, it is possible to see the own institution, the entire city or even a whole state.

Relevant functionalities of IVENA include:

- EMS point of view: This function shows the current status of all hospitals with the intention to support disposition decisions of emergency patients to hospitals through the EMS.
- Notification of hospital closures: This function enables hospital specialty areas to interrupt arrivals of ambulances depending on the level of pre-hospital triage and the subsequent adequate treatment capacity.

The IVENA data contains patient-transport-related data such as injury or disease, condition, age and estimated arrival time as well as assigned hospital. In addition, IVENA data was accumulated to calculate hospital closure duration statistics including reasons for closure.

The goal of the performed simulation model is to suggest improvements for the current situation in the hospitals in Munich via a specific set of policies. The only current limit is a maximum of 24 h closure duration. Seven random consecutive days were selected and the closing times transferred to schedule objects in AnyLogic. These schedules serve as timetables for the availability of service objects (consisting of a queue and a delay) representing the subspecialty unit with a specifically defined delay. When a patient arrives at the closed unit, he gets diverted to the geographically closest hospital that has the same subspecialty unit.

In case the second hospital is closed too, the patient gets diverted to a virtual “other” hospital that represents all remaining hospitals which are not considered in this model. This is based on the assumption, that diversion hardly has more than one iteration, and in case a second diversion is necessary, the patient may get diverted to a smaller but closer hospital than the few biggest hospitals considered here.

The patients arrive according to their original arrival times derived from IVENA (2476 patients, ~ 14 arrivals/h within the observed time period). The patients get triaged a condition according to the condition’s relative frequency and subsequently is assigned to a hospital. During the experiment, parameters such as the number of treated patients, number of diverted patient and closure times are collected. With this data, it will be possible to reenact processes in the department before, during and after a closure.

Following this pre-testing, two simulation models of ambulance diversion are applied. Emergency patients arriving by ambulance are considered, making up around 20% of all patients in the hospitals’ ERs. The first model simulates the current state to understand the system dynamics such as the mean utilized capacity prior, during and after the closure. In the second model, the duration of closures is manipulated to achieve insights on the influence of diversion policies towards the outcome variables. The outcome of the experimental modeling is measured by specific variables captured during the simulation runs such as the number of treated patients, diverted patients, forced assignments, time spent in diversion state, time between two diversion states as well as utilized capacity.

The simulation experiments are performed using AnyLogic 8.1 by The AnyLogic Company.

The individual departments are assigned an upper capacity limit implemented as resource pools of the service element in AnyLogic, determined by the highest number of patients staying in the respective department at the same time during preliminary testing. Three policies were implemented:

- Policy 1: Closure when capacity is reached until patient load is under a threshold (crowding index of 10.8, 0.6 representing 100, 80 and 60% of full capacity).
- Policy 2: Closure for 612, 24 h when capacity is reached.
- Policy 3: Closure for 612, 24 h at CI of 0.8 (80% of full capacity).

*Policy 1* uses the crowding index (CI) as a measure for crowdedness in the unit. The CI is defined by ${\text{CI}} = \frac{\text{current loading of unit}}{\text{capacity of unit}}$. A CI of 1, therefore, implies that a unit is occupied to its full capacity, whereas at a CI of 0.8, 80% of the capacity is occupied. In policy 1, the unit closes once the maximum capacity is reached and opens again when the number of patients in the unit is below the threshold.

*Policy 2* defines specific durations of time periods for which the unit closes, once the full capacity has been reached. The closing time ranges are 6, 12 and 24 h. These proposed closing times are derived from the actual closing times provided by IVENA (mean of 5.8 h).

*Policy 3* intervenes earlier than policy 2 when a CI of 0.8 is reached. All other specifications remain identical.

An overview of these policies is given in Table 1.

**Table 1:** Overview of closing policies and abbreviations

Policy	Abbreviations	Specifications
Policy 1	P1 100 P1 100%	Close when CI = 1 and reopen when CI < 1
	P1 80 P1 80%	Close when CI = 1 and reopen when CI = 0.8
	P1 60 P1 60%	Close when CI = 1 and reopen when CI = 0.6
Policy 2	P2 6 P2 6 h	Close when CI = 1 and reopen after 6 h
	P2 12 P2 12 h	Close when CI = 1 and reopen after 12 h
	P2 24 P2 24 h	Close when CI = 1 and reopen after 24 h
Policy 3	P3 80 6 P3 80% 6 h	Close when CI = 0.8 and reopen after 6 h
	P3 80 12 P3 80% 12 h	Close when CI = 0.8 and reopen after 12 h
	P3 80 24 P3 80% 24 h	Close when CI = 0.8 and reopen after 24 h

Some subspecialty units are in higher demand than others as shown in Table 2. 76.56% of patients are assigned to either general internal medicine, trauma surgery, neurology, pediatrics or the chest pain unit. For simplification reasons, only the five most assigned types of subspecialty units are considered in this simulation model.

**Table 2:** Frequency of assignment to subspecialty units

Subspecialty unit	Absolute frequency (%)	Relative frequency (%)	Covered patients in this subspecialty unit (%)
General internal medicine	37.04	48.38	50.4
Trauma surgery	28.42	37.12	49.95
Neurology	4.16	5.43	96.91
Pediatrics	3.59	4.69	100
Chest pain unit	3.34	4.37	53.94
Total	76.56	100	

Out of each subspecialty area, the five most frequented subspecialty units (four in pediatrics) are considered. The percentage ratio of patients covered by these top five (or four) units is shown in column four. Only four hospitals in Munich have a pediatrics department, therefore, 100% of all pediatric patients are assigned to either one of these hospitals. Overall this simulation model represents 42.28% of all patients. This value was calculated by multiplying the column “absolute frequency” with the percentage of covered patients.

For each triage category, a virtual sixth hospital (or fifth in case of pediatrics) was simulated which receives all patients not covered by the observed units. This approach was taken to keep the number of treated patients in the observed units as close to the real numbers as possible to ensure integrity of this simulation model.

The arrival of patients into the system is based on the original arrival times derived from the IVENA data. Using the database feature of AnyLogic, an Excel sheet containing arrival times was implemented. From this AnyLogic automatically creates agents at the respective point in time. In the observed time period (7 days in January 2016), 2476 patients were assigned to hospitals in Munich.

The length of stay in each subspecialty unit was determined using the outcomes of Cochran and Bharti on the different distributions of length of stay in different units [14]. The authors distinguish between emergency and non-emergency patients. For this simulation model, we are using the distribution of emergency patients as shown in Table 2.

## Results

A total of 536,399 patients were assigned via the IVENA system between February 2013 and March 2017 of whom 1.79% were diverted to a hospital other than their original destination. The increase in the number of patients assigned via IVENA between February 2013 and March 2017 is 17.57%. During the same time, the number of residents of the city of Munich officially increased by 7.07%.

Most patients were assigned in the month of March. However, no seasonal trends were identifiable. The weekday with the most patient assignments is Monday, the one with the least assignments is Sunday. There is a clear increased emergence in the beginning of the week after the weekend, decreasing until Wednesday and then rising until Friday to consequently decrease again during the weekend.

When requesting a closure on the IVENA system, the hospital is required to state a reason. The most frequent reason is “*overcrowding*”, followed by “*not categorized*” which translates into an individual text inserted by the dispatcher.

**Fig. 1:**
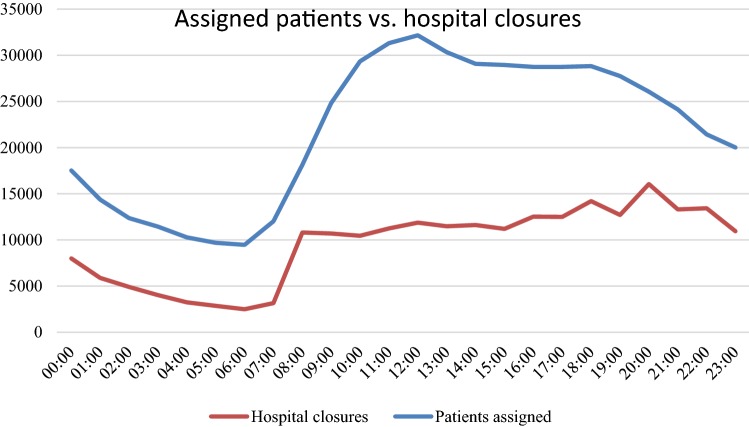
Assigned patients vs hospital closures

Figure 1 shows the development of the number of assigned patients in relation to the number of hospital closures over the course of a day. Both curves show the total amount of assignments and closures, respectively. The brown “closure”-curve clearly follows the shape of the blue “assigned-patients”-curve with a time delay of approximately 1 h. The brown curve states relatively high numbers as it represents all closures, even those of sub departments and specific interventional units such as, e.g., catheter labs or trauma bays.

The steepest increase occurs around 7–8 o’clock in the morning. In the afternoon, both curves stagnate and decrease again in the evening. Figure 1 shows hospital closures as a direct yet delayed consequence of increased patient load. In this analysis, the increase before the closure is quantified and characterized. There is a clear indication that hospital closures are triggered by a sudden increase in patients arriving via EMS. A mean closing time of 308 min (95% confidence interval (CI) [288 min; 329 min]) and a mean interval time between closings of 1101 min (95% CI [1010 min; 1192 min]) was found.

In the Munich area, no common policies exist on which patient load is considered appropriate to justify a closure.

Consequently, several diversion policies were tested in the simulation. Policy 2 and 3 render a fixed time window for closure before the departments open again. Policy 1 reopens once the number of patients in the departments falls below a predefined threshold.

### Simulation outcomes

**Fig. 2:**
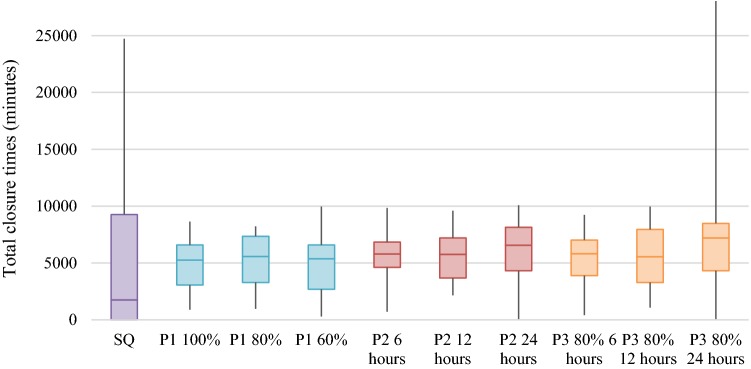
Comparison of policies: boxplot overview of closure times

For both patient and hospital, a short overall closing time as well as a high rate of treated versus diverted patients is desirable. Table 1 shows the specifications of the employed policies. The following graphs show the results of the comparison of the tested policies in regard to these parameters:

Figure 2 shows a boxplot diagram of mean total closure times of the departments under the respective policy. The first boxplot shows the status quo (SQ), derived from the actual closure times during the observed time periods. The rest of the plot only shows little variability in the mean of the total closure times. P1 100% shows the lowest median of all the policies. P3 80% 24 h shows a high variability in total closure hours among the departments.

**Fig. 3:**
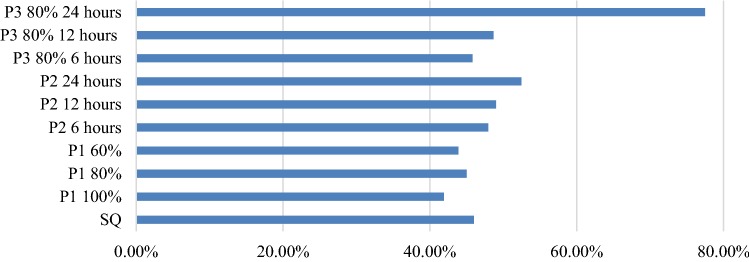
Comparison of policies: mean closure times (percentage)

Figure 3 shows the mean total closure times of all policies as a percentage of the whole simulated time periods. All variations of policy one as well as P3 80% 6 h yield mean total closure times below the mean total closure time of the status quo. Therefore, these four policies could qualify as a proposed improvement of the status quo.

Regardless of the closure time, the most important parameters of all tested policies is the number of treated patients versus the number of diverted patients. Table 3 shows the mean number of treated and diverted patients per policy tested. The lowest possible ratio of diverted to treated patients is desirable. P1 100% is the policy with the lowest diversion/treatment ratio, therefore, yielding most patients treated and least patients diverted. For each patient treated under policy P1 100%, 1.46 patients were diverted.

**Table 3:** Comparison of policies: mean patients treated vs diverted

	P1 100%	P1 80%	P1 60%	P2 6 h	P2 12 h	P2 24 h	P3 80% 6 h	P3 80% 12 h	P3 80% 24 h
Mean treated	20.75	20.38	18.29	19.75	20.04	16.25	18.08	16.50	15.33
Mean diverted	30.29	34.17	41.67	31.42	42.25	49.25	40.63	41.63	50.17
Ratio diverted/treated	1.46	1.68	2.28	1.59	2.11	3.03	2.25	2.52	3.27

## Discussion

Hospital closure and related ambulance diversion has been subject to extensive research and thus been widely discussed [15, 16]. Various approaches have attempted to limit ambulance diversion [11, 17], a multitude of potential solutions was introduced to reduce emergency department crowding [18]. Our findings from the data analysis suggest that ambulance diversion and hospital closures are frequent and their causes foreseeable. As described in the literature, closures pose a disadvantage, potentially even threat to patient safety as well as a significant economic downside for the healthcare system [15]. In the city of Munich, the amount of emergency patients arriving by ambulance is growing proportionally faster than the population. A high percentage of hospital closures is caused by internal capacity problems such as capacity overload, shortage of beds and overcrowding. Via thorough data analyses, every closure can be proven as a direct response to an increased patient load with a delay of approximately 2 h. Previous literature has indicated the general problem and outlined downside effects [19] of closures and diversions. In addition, strategies on relieving ED crowding have been discussed [20]. The underlying study has evaluated more than half a million assignments and thus allows for new insights into the specific approach towards overcrowding via ambulance diversion.

Hoot et al. identify input, throughput or output factors as causes for hospital crowding [21]. Input factors are reasons for a patient’s decision to visit a hospital and include non-urgent visits. Throughput factors are factors prolonging the duration of stay of a patient in the hospital such as inadequate staffing or insufficient resources. Output factors are factors preventing the patient from leaving a hospital department causing delays in inpatient boarding via hospital bed shortages. Furthermore, systematic approaches to prevent emergency department (ED) crowding have been described and suggested as a counteracting method after thorough pattern analysis [22]. Especially, in light of the common knowledge of intra-day-specific differences in degree of crowdedness, this sheds an interesting light into solving organizational difficulties.

A study among patients with acute myocardial infarction (AMI), a time-sensitive condition, found that if the closed ED was on diversion for at least 12 h, the 30-day, 90-day, 9-month and 1-year mortality increased [23]. Liu et al. found that when an ED is on diversion status, other EDs in the same service area are facing a 5% increased mortality rate [24]. The mortality rate increased even more if the diverted patients had time-sensitive conditions such as AMI, strokes or sepsis. A study investigating pediatric mortality could not find an association between diversion and mortality [25].

Several sources put the reduction of ambulance diversion hours into practice by implementing protocols allowing units to go on diversion only under certain circumstances [26, 27] or by setting target maximum hours for diversion times [28]. These measures led to a 73–82% decrease of diversion hours. Following up on the successful reduction of diversion hours by 74% found in the Sacramento study [27, 29] further protocols incorporating a web-based, region-wide emergency medical service software were introduced, similar to IVENA in Munich. As a result, diversion hours could be reduced by 87.4% annually while completely eliminating diversion hours in one county.

Other measures to reduce excessive diversion hours include the implementation of additional resources [30], the introduction of high-turnover utility beds [31] and utilization of web-based information systems [3]. In addition, modern wearable technologies and their effects on ED crowding have been examined. In a total of more than 50,000 patient encounters, however, limits to this technological approach were revealed showing clear limits of innovative technical solutions.

In summary, ambulance diversion is a frequent issue in hospitals worldwide leading to significant effects on patients’ condition. With the patients’ best interest in mind, a better tool for system-wide distribution of patients to counteract bottleneck times is necessary. The presented simulation research results show the necessity for strict diversion criteria. As shown, using past data and artificial intelligence bottlenecks can be prevented and allocation be optimized. As shown in earlier research, in addition the complete medical treatment workflow can be supported by telemedicine [32]. The combination of these innovative technologically supported technologies can help in optimizing medical treatment and reduce redundancies as well as delays in the interest of patient and hospital.

## Limitations

The IVENA data on status and triage is not matched with the latter estimation and treatment in the hospital. Therefore, no follow-up information on the patients, for example length of stay or mortality is available. Further studies may be able to render additional information.

## Conclusion

Hospitals and EMS will need to develop a common set of rules to define closure status prerequisites as one part of an effective solution against overcrowding. A deep understanding of the long-term effects of closure periods as well as a dynamic reopening time may be helpful in optimizing patient allocation and reducing closure periods as well as duration.
